# Bis(*N*-adamantyl-*N*′-ethyl­imidazolium) tetra­bromido­manganate(II)

**DOI:** 10.1107/S2414314620002618

**Published:** 2020-03-03

**Authors:** Niels Ole Giltzau, Martin Köckerling

**Affiliations:** a Universität Rostock, Institut für Chemie, Anorganische Festkörperchemie, Albert-Einstein-Str. 3a, D-18059 Rostock, Germany; b Department Life, Light and Matter, Universität Rostock, 18051 Rostock, Germany; Vienna University of Technology, Austria

**Keywords:** crystal structure, tetra­bromido­manganate, imidazolium, adamant­yl, *N*-adamantyl-*N*′-ethyl-imidazolium, manganese

## Abstract

The title compound, (C_15_H_23_N_2_)_2_[MnBr_4_], comprises two *N*-adamantyl-*N*′-ethyl­imidazolium cations and one tetra­hedral [MnBr_4_]^2−^ anion. Next to Coulombic inter­actions, weak hydrogen bonds of the type C—H⋯Br consolidate the crystal packing, building up a three-dimensional network.

## Abstract 2

The title compound consists of a manganese(II) ion that is bonded to four bromide atoms in a tetra­hedral coordination, and a cation based on an imidazolium group with one ethyl and one adamantyl (the simplest diamondoid) group.

## Graphical abstract

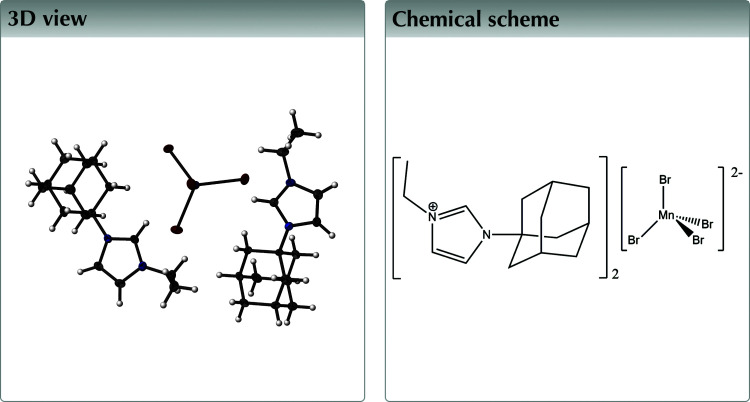

## Structure description

Compounds comprising the tetra­bromido­manganate(II) anion [MnBr_4_]^2–^ are well known and may find applications as green-light-emitting diodes (Xu *et al.*, 2017 ▸). The title compound is a further member of the so far small group of manganese complexes with imidazolium cations (Del Sesto *et al.*, 2008 ▸; Peppel *et al.*, 2019 ▸). To the best of our knowledge, no other structure of a complex salt has been published so far that contains the *N*-adamantyl-*N*′-ethyl-imidazolium cation. Nevertheless, several compounds with adamantyl-imidazolium units have been described to be useful in anion-exchange membranes (Wang *et al.*, 2018 ▸). Compounds containing the adamantyl-substituted imidazolium cation, for which structures have been established, contain two adamantyl (Ad) units, *e.g.* [(Ad)_2_Im]^+^ (Arduengo *et al.*, 1991 ▸; Grasa *et al.*, 2004 ▸).

**Figure 1:**
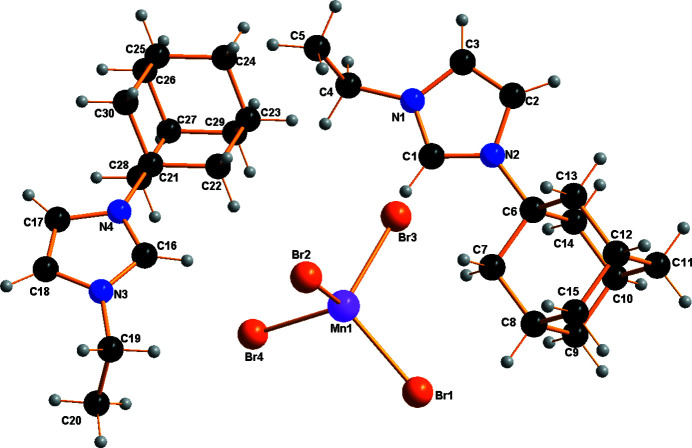
The asymmetric unit of (C_15_H_23_N_2_)_2_[MnBr_4_] with atom labelling.

**Figure 2:**
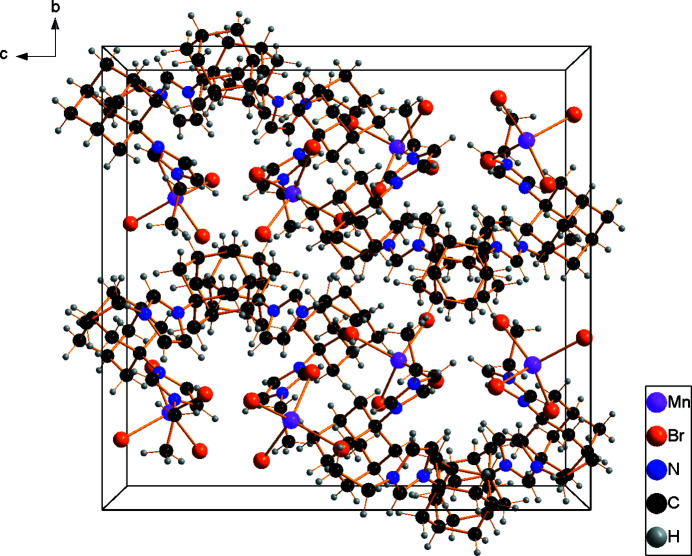
A view of the unit-cell contents in projection down the *a* axis.

**Figure 3:**
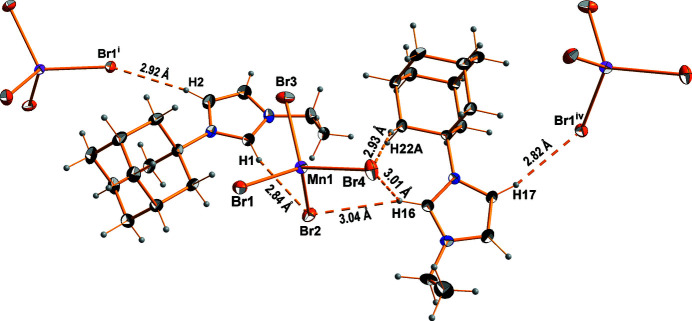
Selected hydrogen bonds between the cations and anions in the crystal structure of (C_15_H_23_N_2_)_2_[MnBr_4_]. Anisotropic displacement ellipsoids are shown at the 50% probability level. Symmetry operators refer to Table 1 ▸.

The asymmetric unit of the title compound comprises two *N*-adamantyl-*N*′-ethyl-imidazolium cations and one tetra­bromido­manganate(II) anion (Fig. 1 ▸). The latter has a slightly distorted tetra­hedral geometry, with the shortest Mn—Br bond length being 2.4983 (6) Å (Mn1—Br4) and the longest 2.5194 (5) Å (Mn1—Br2). The Mn—Br bond lengths are in good agreement with reference values (Orpen *et al.*, 1989 ▸). The Br—Mn—Br angles range from 105.88 (2)° for Br4—Mn1—Br2 to 113.61 (2)° for Br1—Mn1—Br2. The mol­ecular entities of the cation, *viz*. the ethyl group, the imidazole ring and the adamantyl group, have normal distances and angles. In the crystal (Fig. 2 ▸), cations and complex anions are linked *via* an intricate network of weak C—H⋯Br hydrogen bonds into a three-dimensional network (Fig. 3 ▸, Table 1 ▸). Br1 is the acceptor of four contacts, Br2 of three, Br3 of one and Br4 of four.

## Synthesis and crystallization

*N*-adamantyl-*N*′-ethyl-imidazolium bromide (0.19 g, 6 mmol) and MnBr_2_·2H_2_O (0.07 g, 3 mmol) were mixed in methanol (3 ml). The mixture was heated for 3 d at 453 K in a sand bath. After cooling to room temperature, a clear beige-coloured solution was obtained. The solvent was partly removed and large light-green crystals were grown through slow diffusion of diethyl ether or ethyl acetate into the solution. The yield was nearly qu­anti­tative. The compound was also accessible through stirring the starting mixture for several hours at ambient temperature. M.p. 501 K.

## Refinement

Crystal data, data collection and structure refinement details are summarized in Table 2 ▸. Nine reflections were omitted from the structure refinement because their intensities were affected by the beam stop. Details can be found in the refine_special_details field in the CIF.

## Supplementary Material

Crystal structure: contains datablock(s) I. DOI: 10.1107/S2414314620002618/wm4124sup1.cif

Structure factors: contains datablock(s) I. DOI: 10.1107/S2414314620002618/wm4124Isup2.hkl

CCDC reference: 1986182

Additional supporting information:  crystallographic information; 3D view; checkCIF report

## Figures and Tables

**Table 1:** Hydrogen-bond geometry (Å, °)

*D*—H⋯*A*	*D*—H	H⋯*A*	*D*⋯*A*	*D*—H⋯*A*
C1—H1⋯Br2	0.95	2.84	3.766 (3)	166
C2—H2⋯Br1^i^	0.95	2.92	3.864 (3)	170
C3—H3⋯Br4^ii^	0.95	2.99	3.785 (3)	143
C5—H5*C*⋯Br4^ii^	0.98	3.00	3.738 (3)	134
C13—H13*A*⋯Br2^iii^	0.99	3.05	3.941 (3)	151
C16—H16⋯Br2	0.95	3.04	3.929 (3)	156
C16—H16⋯Br4	0.95	3.01	3.549 (3)	117
C17—H17⋯Br1^iv^	0.95	2.82	3.719 (3)	159
C18—H18⋯Br3^v^	0.95	2.82	3.718 (3)	158
C19—H19*A*⋯Br1^v^	0.99	2.81	3.781 (3)	168
C22—H22*A*⋯Br4	0.99	2.93	3.898 (3)	166
C28—H28*A*⋯Br1^iv^	0.99	3.00	3.939 (3)	160

Symmetry codes: (i) 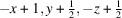 ; (ii) 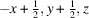 ; (iii) 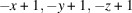 ; (iv) 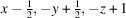 ; (v) 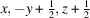 .

**Table 2:** Experimental details

Crystal data	
Chemical formula	(C_15_H_23_N_2_)_2_[MnBr_4_]
*M*_r_	837.29
Crystal system, space group	Orthorhombic, *P* *b* *c* *a*
Temperature (K)	123
*a*, *b*, *c* (Å)	18.020 (1), 18.742 (1), 19.740 (1)
*V* (Å^3^)	6666.6 (7)
*Z*	8
Radiation type	Mo *K*α
μ (mm^−1^)	5.22
Crystal size (mm)	0.22 × 0.22 × 0.05

Data collection	
Diffractometer	Bruker *APEX* KappaII CCD
Absorption correction	Multi-scan (*SADABS*; Krause *et al.*, 2015 ▸)
No. of measured, independent and observed [*I* > 2σ(*I*)] reflections	182157, 5883, 5029
*R*_int_	0.084
(sin θ/λ)_max_ (Å^−1^)	0.595

Refinement	
*R*[*F* ^2^ > 2σ(*F* ^2^)], *wR*(*F* ^2^), *S*	0.027, 0.066, 1.21
No. of reflections	5883
No. of parameters	354
H-atom treatment	H-atom parameters constrained
Δρ_max_, Δρ_min_ (e Å^−3^)	0.72, −0.50

Computer programs: *APEX2* and *SAINT* (Bruker, 2017 ▸), *SHELXT* (Sheldrick, 2015*a* ▸), *SHELXL* (Sheldrick, 2015*b* ▸), *DIAMOND* (Brandenburg & Putz, 2019 ▸) and *publCIF* (Westrip, 2010 ▸).

## full crystallographic data

### Crystal data

**Table:**

(C_15_H_23_N_2_)_2_[MnBr_4_]	*D*_x_ = 1.668 Mg m^−^^3^
*M**_r_* = 837.29	Mo *K*α radiation, λ = 0.71073 Å
Orthorhombic, *P**b**c**a*	Cell parameters from 9879 reflections
*a* = 18.020 (1) Å	θ = 2.4–28.3°
*b* = 18.742 (1) Å	µ = 5.22 mm^−^^1^
*c* = 19.740 (1) Å	*T* = 123 K
*V* = 6666.6 (7) Å^3^	Irregular block, green
*Z* = 8	0.22 × 0.22 × 0.05 mm
*F*(000) = 3352	

### Data collection

**Table:**

Bruker APEX KappaII CCD diffractometer	5029 reflections with *I* > 2σ(*I*)
Radiation source: sealed tube	*R*_int_ = 0.084
φ and ω scans	θ_max_ = 25.0°, θ_min_ = 3.1°
Absorption correction: multi-scan (*SADABS*; Krause *et al*., 2015)	*h* = −21→21
	*k* = −22→22
182157 measured reflections	*l* = −23→23
5883 independent reflections	

### Refinement

**Table:**

Refinement on *F*^2^	Primary atom site location: structure-invariant direct methods
Least-squares matrix: full	Secondary atom site location: difference Fourier map
*R*[*F*^2^ > 2σ(*F*^2^)] = 0.027	Hydrogen site location: inferred from neighbouring sites
*wR*(*F*^2^) = 0.066	H-atom parameters constrained
*S* = 1.21	*w* = 1/[σ^2^(*F*_o_^2^) + (0.0237*P*)^2^ + 8.9922*P*] where *P* = (*F*_o_^2^ + 2*F*_c_^2^)/3
5883 reflections	(Δ/σ)_max_ = 0.001
354 parameters	Δρ_max_ = 0.72 e Å^−^^3^
0 restraints	Δρ_min_ = −0.50 e Å^−^^3^

### Special details

**Table:**

Geometry. All e.s.d.'s (except the e.s.d. in the dihedral angle between two l.s. planes) are estimated using the full covariance matrix. The cell e.s.d.'s are taken into account individually in the estimation of e.s.d.'s in distances, angles and torsion angles; correlations between e.s.d.'s in cell parameters are only used when they are defined by crystal symmetry. An approximate (isotropic) treatment of cell e.s.d.'s is used for estimating e.s.d.'s involving l.s. planes.

### Fractional atomic coordinates and isotropic or equivalent isotropic displacement parameters (Å^2^)

**Table:**

	*x*	*y*	*z*	*U*_iso_*/*U*_eq_	
Mn1	0.40086 (3)	0.31017 (2)	0.38565 (2)	0.0165 (1)	
Br1	0.49923 (2)	0.26519 (2)	0.30615 (2)	0.02108 (8)	
Br2	0.45216 (2)	0.37029 (2)	0.48975 (2)	0.02183 (9)	
Br3	0.32660 (2)	0.40185 (2)	0.32299 (2)	0.02521 (9)	
Br4	0.32464 (2)	0.20800 (2)	0.42767 (2)	0.02693 (9)	
N1	0.3626 (2)	0.5761 (1)	0.4061 (1)	0.0195 (6)	
C1	0.4241 (2)	0.5403 (2)	0.3913 (2)	0.0175 (7)	
H1	0.4393	0.4969	0.4120	0.021*	
N2	0.4607 (1)	0.5746 (1)	0.3433 (1)	0.0169 (6)	
C2	0.4208 (2)	0.6354 (2)	0.3273 (2)	0.0252 (8)	
H2	0.4342	0.6703	0.2946	0.030*	
C3	0.3598 (2)	0.6362 (2)	0.3664 (2)	0.0269 (8)	
H3	0.3220	0.6715	0.3665	0.032*	
C4	0.3072 (2)	0.5525 (2)	0.4560 (2)	0.0257 (8)	
H4A	0.3149	0.5013	0.4658	0.031*	
H4B	0.2570	0.5580	0.4363	0.031*	
C5	0.3115 (2)	0.5940 (2)	0.5210 (2)	0.0256 (8)	
H5A	0.3628	0.5942	0.5375	0.038*	
H5B	0.2793	0.5718	0.5550	0.038*	
H5C	0.2952	0.6432	0.5130	0.038*	
C6	0.5299 (2)	0.5498 (2)	0.3095 (2)	0.0169 (7)	
C7	0.5569 (2)	0.4811 (2)	0.3433 (2)	0.0187 (7)	
H7A	0.5668	0.4900	0.3919	0.022*	
H7B	0.5182	0.4438	0.3398	0.022*	
C8	0.6278 (2)	0.4557 (2)	0.3085 (2)	0.0201 (7)	
H8	0.6456	0.4109	0.3307	0.024*	
C9	0.6113 (2)	0.4407 (2)	0.2337 (2)	0.0223 (7)	
H9A	0.6569	0.4239	0.2106	0.027*	
H9B	0.5731	0.4029	0.2299	0.027*	
C10	0.5835 (2)	0.5092 (2)	0.2001 (2)	0.0242 (8)	
H10	0.5728	0.4997	0.1512	0.029*	
C11	0.6438 (2)	0.5669 (2)	0.2057 (2)	0.0284 (8)	
H11A	0.6895	0.5507	0.1824	0.034*	
H11B	0.6266	0.6113	0.1836	0.034*	
C12	0.6606 (2)	0.5814 (2)	0.2805 (2)	0.0249 (8)	
H12	0.6999	0.6189	0.2842	0.030*	
C13	0.5895 (2)	0.6073 (2)	0.3154 (2)	0.0206 (7)	
H13A	0.5996	0.6174	0.3637	0.025*	
H13B	0.5720	0.6519	0.2937	0.025*	
C14	0.5133 (2)	0.5353 (2)	0.2349 (2)	0.0224 (7)	
H14A	0.4739	0.4988	0.2309	0.027*	
H14B	0.4956	0.5796	0.2128	0.027*	
C15	0.6877 (2)	0.5128 (2)	0.3142 (2)	0.0254 (8)	
H15A	0.7337	0.4961	0.2918	0.030*	
H15B	0.6989	0.5220	0.3625	0.030*	
N3	0.3189 (1)	0.2326 (1)	0.6412 (1)	0.0188 (6)	
C16	0.2941 (2)	0.2756 (2)	0.5933 (2)	0.0170 (7)	
H16	0.3227	0.2938	0.5567	0.020*	
N4	0.2229 (1)	0.2893 (1)	0.6046 (1)	0.0176 (6)	
C17	0.2018 (2)	0.2531 (2)	0.6622 (2)	0.0241 (7)	
H17	0.1538	0.2532	0.6821	0.029*	
C18	0.2615 (2)	0.2178 (2)	0.6850 (2)	0.0244 (8)	
H18	0.2638	0.1883	0.7240	0.029*	
C19	0.3932 (2)	0.2006 (2)	0.6441 (2)	0.0238 (7)	
H19A	0.4137	0.2065	0.6903	0.029*	
H19B	0.4264	0.2259	0.6121	0.029*	
C20	0.3911 (2)	0.1228 (2)	0.6265 (2)	0.0339 (9)	
H20A	0.3746	0.1171	0.5795	0.051*	
H20B	0.3565	0.0981	0.6568	0.051*	
H20C	0.4409	0.1023	0.6317	0.051*	
C21	0.1721 (2)	0.3319 (2)	0.5610 (2)	0.0151 (6)	
C22	0.2154 (2)	0.3683 (2)	0.5044 (2)	0.0184 (7)	
H22A	0.2416	0.3320	0.4769	0.022*	
H22B	0.2528	0.4010	0.5240	0.022*	
C23	0.1614 (2)	0.4104 (2)	0.4596 (2)	0.0200 (7)	
H23	0.1895	0.4342	0.4223	0.024*	
C24	0.1223 (2)	0.4669 (2)	0.5024 (2)	0.0234 (7)	
H24A	0.1594	0.5001	0.5218	0.028*	
H24B	0.0878	0.4948	0.4737	0.028*	
C25	0.0792 (2)	0.4307 (2)	0.5594 (2)	0.0223 (7)	
H25	0.0535	0.4678	0.5873	0.027*	
C26	0.0218 (2)	0.3798 (2)	0.5297 (2)	0.0263 (8)	
H26A	−0.0139	0.4068	0.5015	0.032*	
H26B	−0.0061	0.3563	0.5667	0.032*	
C27	0.0606 (2)	0.3233 (2)	0.4863 (2)	0.0250 (7)	
H27	0.0228	0.2903	0.4666	0.030*	
C28	0.1149 (2)	0.2810 (2)	0.5303 (2)	0.0198 (7)	
H28A	0.0876	0.2562	0.5669	0.024*	
H28B	0.1404	0.2445	0.5024	0.024*	
C29	0.1040 (2)	0.3593 (2)	0.4292 (2)	0.0245 (8)	
H29A	0.0695	0.3861	0.3995	0.029*	
H29B	0.1293	0.3227	0.4014	0.029*	
C30	0.1326 (2)	0.3882 (2)	0.6044 (2)	0.0198 (7)	
H30A	0.1047	0.3646	0.6414	0.024*	
H30B	0.1696	0.4207	0.6250	0.024*	

### Atomic displacement parameters (Å^2^)

**Table:**

	*U*^11^	*U*^22^	*U*^33^	*U*^12^	*U*^13^	*U*^23^
Mn1	0.0176 (3)	0.0162 (2)	0.0156 (2)	−0.0006 (2)	0.0003 (2)	−0.0033 (2)
Br1	0.0200 (2)	0.0267 (2)	0.0165 (2)	0.0050 (1)	0.0003 (1)	−0.0028 (1)
Br2	0.0295 (2)	0.0212 (2)	0.0148 (2)	−0.0047 (1)	−0.0028 (1)	−0.0020 (1)
Br3	0.0272 (2)	0.0236 (2)	0.0249 (2)	0.0070 (1)	−0.0093 (1)	−0.0060 (1)
Br4	0.0278 (2)	0.0201 (2)	0.0330 (2)	−0.0077 (1)	0.0104 (2)	−0.0088 (1)
N1	0.020 (2)	0.017 (1)	0.022 (1)	−0.003 (1)	0.004 (1)	−0.002 (1)
C1	0.022 (2)	0.013 (2)	0.018 (2)	0.000 (1)	0.000 (1)	−0.001 (1)
N2	0.021 (1)	0.014 (1)	0.016 (1)	0.000 (1)	0.002 (1)	0.000 (1)
C2	0.030 (2)	0.019 (2)	0.026 (2)	0.005 (1)	0.005 (2)	0.006 (1)
C3	0.029 (2)	0.021 (2)	0.031 (2)	0.008 (2)	0.005 (2)	0.003 (2)
C4	0.025 (2)	0.021 (2)	0.032 (2)	−0.003 (1)	0.010 (2)	0.001 (1)
C5	0.022 (2)	0.032 (2)	0.022 (2)	−0.001 (2)	0.002 (1)	0.007 (2)
C6	0.021 (2)	0.015 (2)	0.015 (2)	0.001 (1)	0.002 (1)	0.000 (1)
C7	0.025 (2)	0.016 (2)	0.015 (2)	0.001 (1)	0.000 (1)	0.002 (1)
C8	0.022 (2)	0.019 (2)	0.020 (2)	0.003 (1)	0.001 (1)	0.000 (1)
C9	0.026 (2)	0.021 (2)	0.020 (2)	0.002 (1)	0.004 (1)	−0.006 (1)
C10	0.033 (2)	0.026 (2)	0.013 (2)	0.005 (2)	0.001 (1)	−0.001 (1)
C11	0.035 (2)	0.027 (2)	0.024 (2)	0.004 (2)	0.012 (2)	0.006 (2)
C12	0.024 (2)	0.022 (2)	0.028 (2)	−0.005 (1)	0.008 (2)	−0.001 (1)
C13	0.026 (2)	0.015 (2)	0.021 (2)	−0.006 (1)	0.002 (1)	−0.001 (1)
C14	0.029 (2)	0.023 (2)	0.015 (2)	0.004 (1)	−0.003 (1)	−0.001 (1)
C15	0.021 (2)	0.032 (2)	0.023 (2)	0.001 (2)	0.001 (1)	−0.001 (1)
N3	0.018 (1)	0.020 (1)	0.019 (1)	0.004 (1)	−0.002 (1)	0.001 (1)
C16	0.017 (2)	0.018 (2)	0.016 (2)	−0.001 (1)	−0.001 (1)	−0.002 (1)
N4	0.016 (1)	0.020 (1)	0.017 (1)	0.001 (1)	0.001 (1)	0.001 (1)
C17	0.020 (2)	0.030 (2)	0.023 (2)	0.002 (2)	0.003 (1)	0.012 (1)
C18	0.027 (2)	0.027 (2)	0.019 (2)	0.002 (2)	0.002 (2)	0.007 (1)
C19	0.020 (2)	0.027 (2)	0.025 (2)	0.006 (1)	−0.007 (1)	−0.003 (1)
C20	0.032 (2)	0.028 (2)	0.043 (2)	0.010 (2)	0.003 (2)	−0.004 (2)
C21	0.013 (2)	0.017 (2)	0.015 (2)	0.003 (1)	−0.001 (1)	0.002 (1)
C22	0.017 (2)	0.020 (2)	0.018 (2)	0.003 (1)	0.005 (1)	0.002 (1)
C23	0.025 (2)	0.018 (2)	0.017 (2)	0.002 (1)	0.004 (1)	0.006 (1)
C24	0.027 (2)	0.016 (2)	0.027 (2)	0.004 (1)	0.003 (2)	0.004 (1)
C25	0.023 (2)	0.020 (2)	0.024 (2)	0.008 (1)	0.007 (1)	0.002 (1)
C26	0.020 (2)	0.031 (2)	0.029 (2)	0.006 (2)	−0.001 (2)	0.011 (2)
C27	0.024 (2)	0.025 (2)	0.027 (2)	−0.004 (2)	−0.009 (2)	0.004 (1)
C28	0.023 (2)	0.016 (2)	0.021 (2)	−0.001 (1)	−0.001 (1)	0.003 (1)
C29	0.034 (2)	0.023 (2)	0.018 (2)	0.004 (2)	−0.004 (2)	0.002 (1)
C30	0.022 (2)	0.020 (2)	0.017 (2)	0.002 (1)	0.005 (1)	0.000 (1)

### Geometric parameters (Å, º)

**Table:**

Mn1—Br4	2.4983 (6)	C15—H15A	0.9900
Mn1—Br3	2.5046 (6)	C15—H15B	0.9900
Mn1—Br1	2.5130 (6)	N3—C16	1.321 (4)
Mn1—Br2	2.5194 (5)	N3—C18	1.375 (4)
N1—C1	1.327 (4)	N3—C19	1.468 (4)
N1—C3	1.373 (4)	C16—N4	1.327 (4)
N1—C4	1.470 (4)	C16—H16	0.9500
C1—N2	1.322 (4)	N4—C17	1.379 (4)
C1—H1	0.9500	N4—C21	1.488 (4)
N2—C2	1.384 (4)	C17—C18	1.340 (5)
N2—C6	1.488 (4)	C17—H17	0.9500
C2—C3	1.343 (5)	C18—H18	0.9500
C2—H2	0.9500	C19—C20	1.501 (5)
C3—H3	0.9500	C19—H19A	0.9900
C4—C5	1.503 (5)	C19—H19B	0.9900
C4—H4A	0.9900	C20—H20A	0.9800
C4—H4B	0.9900	C20—H20B	0.9800
C5—H5A	0.9800	C20—H20C	0.9800
C5—H5B	0.9800	C21—C22	1.524 (4)
C5—H5C	0.9800	C21—C28	1.529 (4)
C6—C13	1.526 (4)	C21—C30	1.534 (4)
C6—C14	1.527 (4)	C22—C23	1.533 (4)
C6—C7	1.531 (4)	C22—H22A	0.9900
C7—C8	1.527 (4)	C22—H22B	0.9900
C7—H7A	0.9900	C23—C24	1.527 (4)
C7—H7B	0.9900	C23—C29	1.531 (5)
C8—C15	1.522 (5)	C23—H23	1.0000
C8—C9	1.532 (4)	C24—C25	1.526 (4)
C8—H8	1.0000	C24—H24A	0.9900
C9—C10	1.530 (4)	C24—H24B	0.9900
C9—H9A	0.9900	C25—C26	1.525 (5)
C9—H9B	0.9900	C25—C30	1.533 (4)
C10—C14	1.520 (5)	C25—H25	1.0000
C10—C11	1.537 (5)	C26—C27	1.530 (5)
C10—H10	1.0000	C26—H26A	0.9900
C11—C12	1.531 (5)	C26—H26B	0.9900
C11—H11A	0.9900	C27—C29	1.529 (5)
C11—H11B	0.9900	C27—C28	1.532 (4)
C12—C15	1.527 (5)	C27—H27	1.0000
C12—C13	1.534 (5)	C28—H28A	0.9900
C12—H12	1.0000	C28—H28B	0.9900
C13—H13A	0.9900	C29—H29A	0.9900
C13—H13B	0.9900	C29—H29B	0.9900
C14—H14A	0.9900	C30—H30A	0.9900
C14—H14B	0.9900	C30—H30B	0.9900
			
Br4—Mn1—Br3	113.33 (2)	C8—C15—H15B	109.8
Br4—Mn1—Br1	109.76 (2)	C12—C15—H15B	109.8
Br3—Mn1—Br1	107.37 (2)	H15A—C15—H15B	108.2
Br4—Mn1—Br2	105.88 (2)	C16—N3—C18	108.6 (3)
Br3—Mn1—Br2	106.98 (2)	C16—N3—C19	125.8 (3)
Br1—Mn1—Br2	113.61 (2)	C18—N3—C19	125.4 (3)
C1—N1—C3	108.6 (3)	N3—C16—N4	108.9 (3)
C1—N1—C4	124.2 (3)	N3—C16—H16	125.5
C3—N1—C4	127.2 (3)	N4—C16—H16	125.5
N2—C1—N1	109.2 (3)	C16—N4—C17	108.1 (3)
N2—C1—H1	125.4	C16—N4—C21	127.0 (3)
N1—C1—H1	125.4	C17—N4—C21	124.8 (3)
C1—N2—C2	107.8 (3)	C18—C17—N4	107.4 (3)
C1—N2—C6	126.0 (3)	C18—C17—H17	126.3
C2—N2—C6	126.1 (3)	N4—C17—H17	126.3
C3—C2—N2	107.6 (3)	C17—C18—N3	107.0 (3)
C3—C2—H2	126.2	C17—C18—H18	126.5
N2—C2—H2	126.2	N3—C18—H18	126.5
C2—C3—N1	106.8 (3)	N3—C19—C20	111.4 (3)
C2—C3—H3	126.6	N3—C19—H19A	109.4
N1—C3—H3	126.6	C20—C19—H19A	109.4
N1—C4—C5	112.4 (3)	N3—C19—H19B	109.4
N1—C4—H4A	109.1	C20—C19—H19B	109.4
C5—C4—H4A	109.1	H19A—C19—H19B	108.0
N1—C4—H4B	109.1	C19—C20—H20A	109.5
C5—C4—H4B	109.1	C19—C20—H20B	109.5
H4A—C4—H4B	107.9	H20A—C20—H20B	109.5
C4—C5—H5A	109.5	C19—C20—H20C	109.5
C4—C5—H5B	109.5	H20A—C20—H20C	109.5
H5A—C5—H5B	109.5	H20B—C20—H20C	109.5
C4—C5—H5C	109.5	N4—C21—C22	110.4 (2)
H5A—C5—H5C	109.5	N4—C21—C28	108.0 (2)
H5B—C5—H5C	109.5	C22—C21—C28	109.5 (2)
N2—C6—C13	109.5 (2)	N4—C21—C30	109.3 (2)
N2—C6—C14	109.0 (3)	C22—C21—C30	109.8 (2)
C13—C6—C14	109.7 (3)	C28—C21—C30	109.8 (3)
N2—C6—C7	109.5 (2)	C21—C22—C23	109.1 (3)
C13—C6—C7	109.7 (3)	C21—C22—H22A	109.9
C14—C6—C7	109.5 (2)	C23—C22—H22A	109.9
C8—C7—C6	109.3 (2)	C21—C22—H22B	109.9
C8—C7—H7A	109.8	C23—C22—H22B	109.9
C6—C7—H7A	109.8	H22A—C22—H22B	108.3
C8—C7—H7B	109.8	C24—C23—C29	109.8 (3)
C6—C7—H7B	109.8	C24—C23—C22	109.3 (3)
H7A—C7—H7B	108.3	C29—C23—C22	109.5 (3)
C15—C8—C7	109.9 (3)	C24—C23—H23	109.4
C15—C8—C9	109.8 (3)	C29—C23—H23	109.4
C7—C8—C9	109.2 (3)	C22—C23—H23	109.4
C15—C8—H8	109.3	C25—C24—C23	109.5 (3)
C7—C8—H8	109.3	C25—C24—H24A	109.8
C9—C8—H8	109.3	C23—C24—H24A	109.8
C10—C9—C8	109.1 (3)	C25—C24—H24B	109.8
C10—C9—H9A	109.9	C23—C24—H24B	109.8
C8—C9—H9A	109.9	H24A—C24—H24B	108.2
C10—C9—H9B	109.9	C26—C25—C24	109.8 (3)
C8—C9—H9B	109.9	C26—C25—C30	108.9 (3)
H9A—C9—H9B	108.3	C24—C25—C30	109.8 (3)
C14—C10—C9	110.3 (3)	C26—C25—H25	109.4
C14—C10—C11	109.2 (3)	C24—C25—H25	109.4
C9—C10—C11	109.1 (3)	C30—C25—H25	109.4
C14—C10—H10	109.4	C25—C26—C27	109.8 (3)
C9—C10—H10	109.4	C25—C26—H26A	109.7
C11—C10—H10	109.4	C27—C26—H26A	109.7
C12—C11—C10	109.6 (3)	C25—C26—H26B	109.7
C12—C11—H11A	109.8	C27—C26—H26B	109.7
C10—C11—H11A	109.8	H26A—C26—H26B	108.2
C12—C11—H11B	109.8	C29—C27—C26	109.9 (3)
C10—C11—H11B	109.8	C29—C27—C28	108.7 (3)
H11A—C11—H11B	108.2	C26—C27—C28	109.5 (3)
C15—C12—C11	109.5 (3)	C29—C27—H27	109.6
C15—C12—C13	109.7 (3)	C26—C27—H27	109.6
C11—C12—C13	108.9 (3)	C28—C27—H27	109.6
C15—C12—H12	109.6	C21—C28—C27	109.4 (2)
C11—C12—H12	109.6	C21—C28—H28A	109.8
C13—C12—H12	109.6	C27—C28—H28A	109.8
C6—C13—C12	109.3 (3)	C21—C28—H28B	109.8
C6—C13—H13A	109.8	C27—C28—H28B	109.8
C12—C13—H13A	109.8	H28A—C28—H28B	108.2
C6—C13—H13B	109.8	C27—C29—C23	109.4 (3)
C12—C13—H13B	109.8	C27—C29—H29A	109.8
H13A—C13—H13B	108.3	C23—C29—H29A	109.8
C10—C14—C6	109.3 (3)	C27—C29—H29B	109.8
C10—C14—H14A	109.8	C23—C29—H29B	109.8
C6—C14—H14A	109.8	H29A—C29—H29B	108.2
C10—C14—H14B	109.8	C25—C30—C21	108.9 (2)
C6—C14—H14B	109.8	C25—C30—H30A	109.9
H14A—C14—H14B	108.3	C21—C30—H30A	109.9
C8—C15—C12	109.5 (3)	C25—C30—H30B	109.9
C8—C15—H15A	109.8	C21—C30—H30B	109.9
C12—C15—H15A	109.8	H30A—C30—H30B	108.3

### Hydrogen-bond geometry (Å, º)

**Table:**

*D*—H···*A*	*D*—H	H···*A*	*D*···*A*	*D*—H···*A*
C1—H1···Br2	0.95	2.84	3.766 (3)	166
C2—H2···Br1^i^	0.95	2.92	3.864 (3)	170
C3—H3···Br4^ii^	0.95	2.99	3.785 (3)	143
C5—H5*C*···Br4^ii^	0.98	3.00	3.738 (3)	134
C13—H13*A*···Br2^iii^	0.99	3.05	3.941 (3)	151
C16—H16···Br2	0.95	3.04	3.929 (3)	156
C16—H16···Br4	0.95	3.01	3.549 (3)	117
C17—H17···Br1^iv^	0.95	2.82	3.719 (3)	159
C18—H18···Br3^v^	0.95	2.82	3.718 (3)	158
C19—H19*A*···Br1^v^	0.99	2.81	3.781 (3)	168
C22—H22*A*···Br4	0.99	2.93	3.898 (3)	166
C28—H28*A*···Br1^iv^	0.99	3.00	3.939 (3)	160

Symmetry codes: (i) −*x*+1, *y*+1/2, −*z*+1/2; (ii) −*x*+1/2, *y*+1/2, *z*; (iii) −*x*+1, −*y*+1, −*z*+1; (iv) *x*−1/2, −*y*+1/2, −*z*+1; (v) *x*, −*y*+1/2, *z*+1/2.
